# Radiomics–clinical nomogram based on pretreatment 18F-FDG PET-CT radiomics features for individualized prediction of local failure in nasopharyngeal carcinoma

**DOI:** 10.1038/s41598-023-44933-7

**Published:** 2023-10-24

**Authors:** Jianming Ding, Zirong Li, Yuhao Lin, Chaoxiong Huang, Jiawei Chen, Jiabiao Hong, Zhaodong Fei, Qichao Zhou, Chuanben Chen

**Affiliations:** 1 Department of Radiation Oncology, Clinical Oncology School of Fujian Medical University, Fujian Cancer Hospital, Fuma Road, FuzhouFujian, 350014 China; 2 Manteia Technologies Co., Ltd, 1903, B Tower, Zijin Plaza, No.1811 Huandao East Road, Xiamen, China

**Keywords:** Radiotherapy, Head and neck cancer

## Abstract

To explore the prognostic significance of PET/CT-based radiomics signatures and clinical features for local recurrence-free survival (LRFS) in nasopharyngeal carcinoma (NPC). We retrospectively reviewed 726 patients who underwent pretreatment PET/CT at our center. Least absolute shrinkage and selection operator (LASSO) regression and the Cox proportional hazards model were applied to construct Rad-score, which represented the radiomics features of PET-CT images. Univariate and multivariate analyses were used to establish a nomogram model. The concordance index (C-index) and calibration curve were used to evaluate the predictive accuracy and discriminative ability. Receiver operating characteristic analysis was performed to stratify the local recurrence risk of patients. The nomogram was validated by evaluating its discrimination ability and calibration in the validation cohort. A total of eight features were selected to construct Rad-score. A radiomics–clinical nomogram was built after the selection of univariate and multivariable Cox regression analyses, including the Rad-score and maximum standardized uptake value (SUVmax). The C-index was 0.71 (0.67–0.74) in the training cohort and 0.70 (0.64–0.76) in the validation cohort. The nomogram also performed far better than the 8th T-staging system with an area under the receiver operating characteristic curve (AUC) of 0.75 vs. 0.60 for 2 years and 0.71 vs. 0.60 for 3 years. The calibration curves show that the nomogram indicated accurate predictions. Decision curve analysis (DCA) revealed significantly better net benefits with this nomogram model. The log-rank test results revealed a distinct difference in prognosis between the two risk groups. The PET/CT-based radiomics nomogram showed good performance in predicting LRFS and showed potential to identify patients at high-risk of developing NPC.

## Introduction

Nasopharyngeal carcinoma (NPC) is a rare cancer with a highly asymmetric geographical distribution. According to the International Agency for Research on Cancer, approximately 129,000 new cases of NPC were diagnosed worldwide in 2018. The majority of new cases were reported in East Asia and Southeast Asia^1–3^. Radiotherapy, with some cases requiring the addition of chemotherapy, is the standard treatment for NPC. Intensity-modulated radiation (IMRT) is a revolutionary radiotherapy technique that has considerably improved the treatment outcomes of patients with NPC, particularly in terms of local control. Although extensive use of IMRT has contributed to excellent local control, local recurrence remains an important cause of treatment failure in ~ 10% of advanced NPC cases. Owing to the potential existence of a disease with intrinsic radioresistance, the salvage treatment options are complex and limited for patients with locally recurrent NPC and typically present with a poor prognosis for patients with rT3–T4 disease^4,5^. Local recurrence plays a particularly vital role in treatment failure. Therefore, a more accurate prediction method is required to identify the individuals who are resistant to treatment and susceptible to recurrence and metastasis. Adjusting the prediction-based therapeutic strategy could provide a more favorable outcome. Although the American Joint Committee on Cancer TNM (AJCC/TNM) staging system is the key to prognosis prediction and risk stratification, the prediction of local failure still can be improved in some ways^6^.

18F-Fluorodeoxyglucose positron emission tomography/computed tomography (18F-FDG PET/CT) imaging is becoming widely implemented because of its better sensitivity and specificity than conventional anatomical imaging techniques like CT^7^. The spectrum of information provided by 18F-FDG PET/CT improves the early detection of cancer and consequently the treatment outcome. Coincidentally, it has excellent utility in combination with radiomics. Radiomics, a relatively new field aimed at connecting medical imaging information with biological and clinical endpoints, has grown into a promising field in oncology. By translating medical imaging into mineable, high-dimensional, and quantitative imaging features via high-throughput extraction of data-characterization algorithms, radiomics provides a brand new and dependable way to strengthen the predictive ability of a prediction model^8–10^. To our knowledge, the predictive capability of 18F-FDG PET/CT features for local failure NPC remains unclear. Thus, this research aimed to establish a radiomics signature based on ^18^F-FDG PET/CT to predict local failure.

## Materials and methods

### Patient

This retrospective study enrolled 726 patients with newly diagnosed NPC who underwent complete treatment at our center between January 2012 and December 2018. We included patients (a) with a biopsy-confirmed primary NPC; (b) with comprehensive baseline clinical data available; (c) with pretreatment whole-body 18F-FDG PET/CT findings available; and (d) having undergone radical treatment. We excluded patients with (a) distant metastasis; (b) other malignancies; (c) a concomitant fatal disease; (d) a history of cancer treatment; and (e) insufficient follow-up data available. All patients were staged according to the 8th edition of the AJCC/TNM staging system. Our study was performed in accordance with the Helsinki Declaration. The Ethics Committee of the Fujian Cancer Hospital authorized this study (YKT2020-011-01).

### Image processing

The whole-body [18F] FDG PET/CT scan before treatment was performed using a Gemini TF-64 PET/CT scanner (Philips, The Netherlands), and 18F-FDG was manufactured by HM-10 cyclotron. Patients fasted for over 6 h to maintain a serum blood glucose level of 3.9–6.5 mmol/L before radiotracer injection. Then, images were acquired 40–60 min after FDG injection at a dose of 148–296 MBq. A low-dose CT from the head to the proximal thigh (2.5 mA, 140 kV, 4-mm slice thickness, matrix: 512 × 512) was obtained for attenuation correction and fusion purposes. PET image reconstructions were finished using the manufacturer’s proprietary Blob-ordered-subset-time-of-flight algorithm.

The entire segmentation process was performed using Accucontour software (Version 3.0, http://www.manteiatech.com/). The primary tumor was delineated on PET-CT imaging by a single observer (clinical radiation therapist, 10 years of experience) under the guidance of an experienced superordinate physician (20 years of experience). Pretreatment enhanced MRI and/or CT images were used as a guide for PET-CT delineation. The ROI of the primary tumor (ROI-P) was semi-automatically segmented based on the region with an SUVmax of more than 2.5. Each ROI was evaluated and manually adjusted, if necessary. The ROI-P of CT was automatically generated from the corresponding PET volumes.

### Feature extraction and reduction

Radiomics takes high-definition images from ROIs and effectively turns medical images into multi-dimensional mineable features by quantifying information on tumor shape, size, volume, texture, and intensity features. For each ROI, about 3375 features were extracted from original and derived images [seven built-in filter features: Laplacian of Gaussian (LOG), wavelet, sigma, square, square root, logarithm, gradient, and exponential], which were obtained from ROI-P in both PET and CT images using the Pyradiomics package (version 2.12). The fixed bin width was set to 0.1 g/mL for PET images, 25 HU for CT images. The features could be categorized as follows: shape-based features, first-order statistics, and texture features [e.g., gray level cooccurrence matrix (GLCM), gray level run length matrix (GLRLM), gray level size zone matrix (GLSZM), gray level dependence matrix (GLDM), and neighboring gray tone difference matrix (NGTDM)].

To increase algorithmic stability and performance, all features were scaled using Z-score normalization. Next, the univariate analysis was first used to select the relevant radiomics features with LRFS (*P* < 0.05). Then, Spearman’s correlation analysis was performed to reduce the redundancy of radiomics features. One of the paired significantly correlated features was removed by completely random (*P* < 0.05; correlation coefficient > 0.9).

### Prediction model construction and evaluation

A radiomics model called Rad-score was built using least absolute shrinkage and selection operator (LASSO) regression and the Cox proportional hazards model for predicting local recurrence-free survival (LRFS) of patients with NPC in the training set. The training cohort was split as follows: 80% of the data represented the verification cohort for parameter selection and model construction and 20% of the data represented the test cohort for model validation. Three-fold cross-validation was performed, and the Rad-score and clinical parameters were subjected to univariate analysis. Then, statistically significant variables were included in multivariable Cox regression analysis to identify independent prognostic factors. Then, the factors identified were used to establish a radiomics–clinical model. A nomogram was constructed to predict 2- and 3-year LRFS rates on the basis of the radiomics–clinical model. The concordance index (C-index) and area under the receiver operating characteristic curve (AUC) analysis were used to evaluate the nomogram's discriminating power. The calibration curve was derived to compare the observed and predicted probabilities. DCA was used to evaluate the clinical validity of the nomogram by quantifying the net benefits at different threshold probabilities. Then, the ROC curve was used to classify the patients into two risk groups. Using the Kaplan–Meier method of survival analysis, survival curves for the different risk groups were plotted and then compared using the log-rank test.

### Treatment

Platinum-based chemotherapy was delivered to 682 patients (682/726, 93.9%). In the entire cohort, 624 (86%), 532 (73.3%), and 162 (22.3%) cases underwent neoadjuvant, concurrent, and adjuvant chemotherapy, respectively. The most commonly used regimen was platinum + paclitaxel/gemcitabine. Intensity-modulated radiotherapy (IMRT) was used for radiotherapy. The target volume and radiotherapy dose were implemented according to a previously described protocol^11^. The prescribed radiotherapy doses were as follows—GTV: 70–72.6 Gy/31–33 fx, CTV1: 62–62.7 Gy/31–33 fx, and CTV2: 54.4–56.2 Gy/31–33 fx.

### Endpoints and follow-up

Notably, LRFS, which was defined as the time from pathological diagnosis to local relapse or the end of follow-up, was selected as the primary endpoint. All patients had a regular follow-up schedule—every 3 months for 2 years, every 6 months for years 3–5, and then annual visits.

### Statistical analysis

All analysis were performed using R and Python. The Chi-squared test was used to validate differences between the two variables. The ROC curve determined the optimal cutoff value for Rad-score, EBV-DNA, SUVmax, and other continuous variables to predict local recurrence with the maximum value of the Youden’s index. The log-rank test was used to assess the statistical significance of differences between the two survival groups in Kaplan–Meier survival analysis. A statistically significant difference was indicated by a two-sided *P* value of < 0.05.

### Ethics approval and consent to participate

The study was approved by the Ethical Committee of Fujian Cancer Hospital (YKT2020-011-01) and was conducted in accordance with the 1964 Helsinki declaration and its later amendments or comparable ethical standards. Patient identifiers, such as names, were not collected, and instead, patients were given a numerical identifier. Informed consent was obtained from all participants, and for those aged under 18 years, the consent was obtained from a parent or legal guardian. For confidentiality, the patients’ charts were used only within the confines of the records department, and only the investigators and study assistant had access to the files.

## Results

### Patient characteristics and survival

A total of 726 patients were recruited for our research. To construct and validate the nomogram, patients were randomly divided into a training cohort (n = 580) and validation cohort (n = 146) in an 8:2 ratio. Table 1 lists the baseline of patients recruited in training and validation cohorts. There were no statistically significant differences between the training set and the validation set (*P* range: 0.090–1.000). The median follow-up times for training and validation groups were 48 months (range: 3–118 months) and 50.5 months (range: 9–118 months), respectively. There were 50 cases of local recurrence in the training group and 13 such cases in the validation group. Furthermore, the 2- and 3-year LRFS rates for the training cohort were 95.4% and 93.6%, respectively. The 2- and 3-year LRFS rates for the validation cohort were 97.3% and 93.1%, respectively. The above information indicates that the patients in the training and validation cohorts had balanced distribution of survival and baseline characteristics.

**Table 1:** Characteristics of patients recruited in training and validation cohorts.

Characteristic	Training set, n (%)	Validation set, n (%)	*P*
N	580 (79.9)	146 (20.1)	
Sex			0.252
Male	415 (71.6)	112 (76.7)	
Female	165 (28.4)	34 (23.3)	
Age			0.503
≤ 44 years	238 (41)	65 (44.5)	
> 44 years	342 (59)	81 (55.5)	
T stage			0.098
1	129 (22.2)	42 (28.8)	
2	122 (21)	28 (19.2)	
3	213 (36.7)	58 (39.7)	
4	116 (20)	18 (12.3)	
N stage			0.090
0	64 (11)	27 (18.5)	
1	204 (35.2)	46 (31.5)	
2	186 (32.1)	47 (32.2)	
3	126 (21.7)	26 (17.8)	
SUVmax			0.760
≤ 7.44	221 (38.1)	53 (36.3)	
> 7.44	359 (61.9)	93 (63.7)	
RS			0.590
≤ 0.27	373 (64.3)	98 (67.1)	
> 0.27	207 (35.7)	48 (32.9)	
SII			0.277
≤ 321.75	102 (17.6)	32 (21.9)	
> 321.75	478 (82.4)	114 (78.1)	
NE (*10^9^/L)			0.812
≤ 3.8	256 (44.4)	67 (45.9)	
> 3.8	321 (55.6)	79 (54.1)	
Lym (*10^9^/L)			0.638
≤ 1.5	107 (18.5)	24 (16.4)	
> 1.5	470 (81.5)	122 (83.6)	
Hb (g/L)			0.807
≤ 148	333 (57.7)	82 (56.2)	
> 148	244 (42.3)	64 (43.8)	
EBV-DNA (copies/mL)			0.111
≤ 34,200	475 (83)	128 (88.9)	
> 34,200	97 (17)	16 (11.1)	
LRFS state			1.000
0	530 (91.4)	133 (91.1)	
1	50 (8.6)	13 (8.9)	

*RS* Rad-score, *SII* systemic immune-inflammation, *NE* neutrophil, *Lym* lymphocytes, *Hb* hemoglobin, *EBV-DNA* plasma Epstein–Barr virus DNA, *SUVmax* maximum standardized uptake value, *LRFS* local recurrence free survival.

### Radiomics signature building

**Figure 1:**
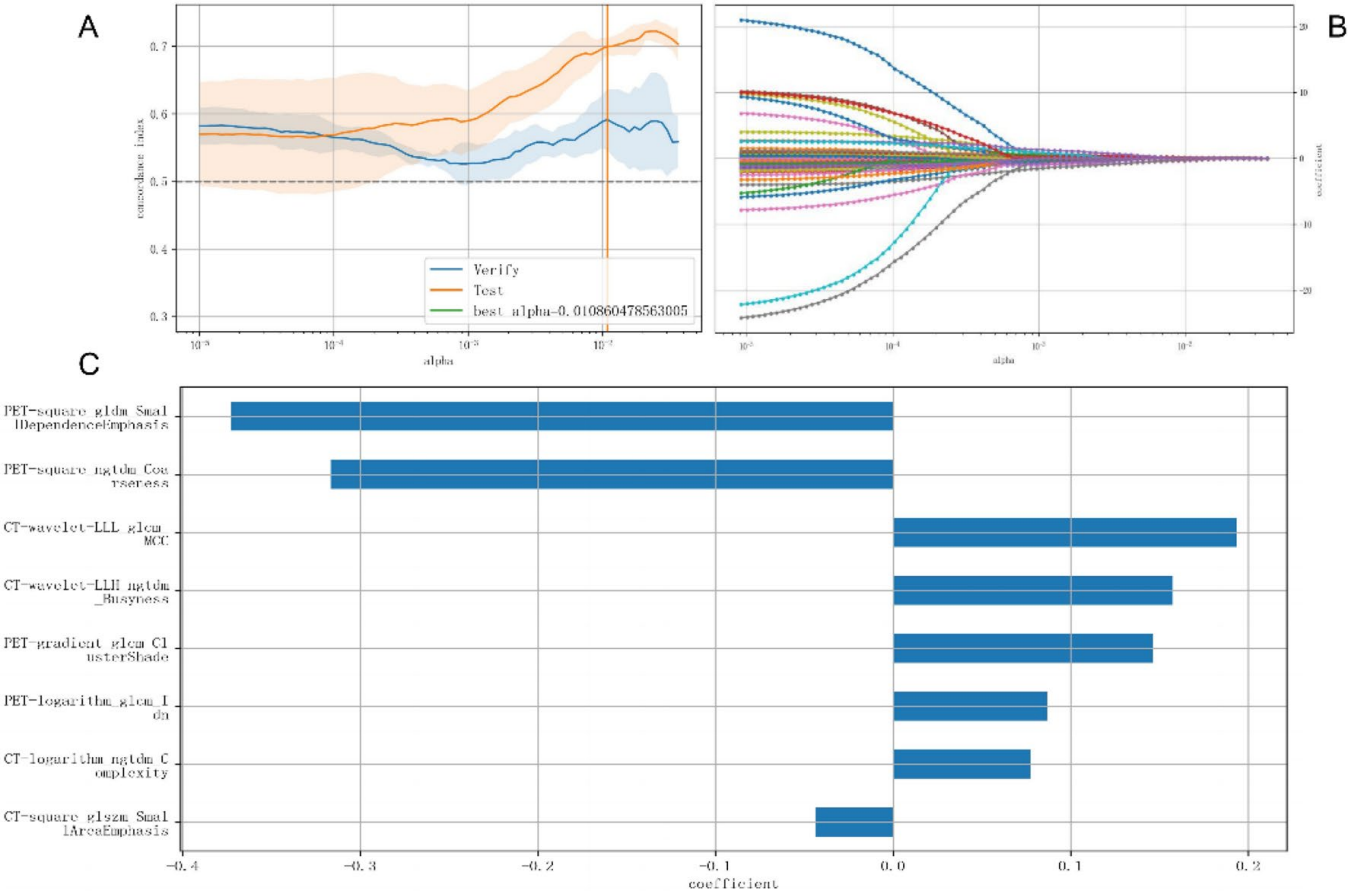
Radiomics signature building. (**A**,**B**) Radiomics feature screened by LASSO regression and the Cox proportional hazards model; (**C**) the contribution of the selected features to build Rad-score and their corresponding regression coefficients.

A total of 85 features remained after dimensionality reduction, which included Spearman’s correlation and univariate analysis. To avoid over-fitting, we used LASSO regression and the Cox proportional hazards model to identify potential features and build the Rad-score model. The α value (0.0108) was determined during cross-validation, which made the C-index of the verification cohort have the highest mean value (0.592, range: 0.547–0.637 in the verification cohort; 0.70, range: 0.688–0.712 in the test cohort). Details of LASSO regression and the Cox proportional hazards model are shown in Fig. 1A,B. We identified eight potential radiomics features, namely PET-gradient_glcm_Clustershade, PET-logarithm_glcm_ldn, PET-square_gldm_SmallDependenceEmphasis, PET-square_ngtdm_Coarseness, CT-wavelet-LLH_ngtdm_Busyness, CT-wavelet-LLL_glcm_MCC, CT-logarithm_ngtdm_Complexity, and CT-square_glszm_SmallAreaEmphasis. Based on the Cox regression analysis, the constructed RAD-score model had a C-index of 0.650 (range: 0.618–0.682) in the training cohort and 0.652 (range: 0.591–0.713) in the validation cohort. The contribution of the selected features to build score and their corresponding regression coefficients are shown in Fig. 1C. These features were linearly combined to get each patient’s Rad-score, which was determined using the formula shown below.

Rad-score = 0.23*PET-gradient_glcm_Clustershade + 0.06*PET-logarithm_glcm_ldn-0.55*PET-square_gldm_SmallDependenceEmphasis-0.54*PET-square_ngtdm_Coarseness + 0.17*CT-wavelet-LLH_ngtdm_Busyness + 0.31*CT-wavelet-LLL_glcm_MCC + 0.11*CT-logarithm_ngtdm_Complexity-0.07*CT-square_glszm_SmallAreaEmphasis.

### Development of radiomics–clinical nomogram

To build a robust prediction model, we enrolled Rad-score and several clinical parameters, including patients’ sex, age, T-stage, N stage, Rad-score (RS), systemic immune-inflammation index (SII), neutrophils (NE), lymphocytes (Lym), hemoglobin (Hb), and plasma Epstein–Barr virus DNA (EBV-DNA). Continuous variables were transformed into categorical variables to get meaningful information on the basis of the optimal cut-off value, which was determined using the maximum Youden’s index. Cut-off values were calculated using the ROC curve, and the specific cut-off values are shown below: age (44 years), RS (0.27), SII (321.75), NE (3.8*10^9^/L), Lym (1.5*10^9^/L) Hb (148 g/L), and EBV-DNA (34,200 copies/mL).

**Figure 2:**
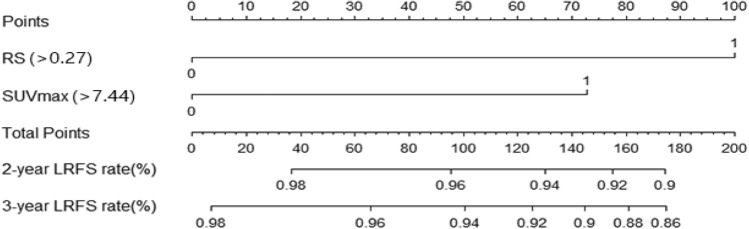
Nomogram model predicting 2- and 3-year local failure in patients with NPC.

In multivariate analysis of LRFS, the results showed that RS (*P* < 0.001) and SUVmax (P < 0.033) were independent indicators of prognosis. The holistic results of univariate and multivariate analyses are shown in Table 2. We integrated the selected independent risk factors to build a comprehensive nomogram for predicting 2- and 3-year LRFS in the training cohort (Fig. 2). The total points gained by multiple variables are related to the predicted probability for a patient. Higher points in the nomogram indicate a shorter LRFS.

**Table 2:** Univariate and multivariate analyses of risk factors for local failure in patients with NPC.

Characteristics	Univariate analysis		Multivariate analysis	
	Hazard ratio (95% CI)	*P* value	Hazard ratio (95% CI)	*P* value
Sex, n (%)				
Male	Reference			
Female	0.853 (0.453–1.605)	0.623		
T stage				
1	Reference			
2	1.624 (0.578–4.563)	0.358	1.225 (0.433–3.463)	0.702
3	2.103 (0.839–5.273)	0.113	1.040 (0.396–2.728)	0.937
4	3.239 (1.264–8.297)	**0.014**	1.057 (0.379–2.948)	0.915
N stage				
0	Reference			
1	4.175 (0.989–17.631)	0.052		
2	2.392 (0.535–10.697)	0.254		
3	3.312 (0.734–14.947)	0.119		
RS				
≤ 0.27	Reference			
> 0.27	4.224 (2.328–7.667)	** < 0.001**	3.171 (1.639–6.136)	** < 0.001**
Age				
≤ 44	Reference			
> 44	1.410 (0.785–2.532)	0.250		
SUVmax				
≤ 7.44	Reference			
> 7.44	3.625 (1.699–7.732)	** < 0.001**	2.419 (1.073–5.455)	**0.033**
EBV-DNA				
≤ 34,200	Reference			
> 34,200	1.709 (0.868–3.364)	0.121		
SII				
≤ 321.75	Reference			
> 321.75	1.351 (0.607–3.004)	0.461		
NE				
≤ 3.8	Reference			
> 3.8	1.519 (0.846–2.728)	0.161		
Lym				
≤ 1.5	Reference			
> 1.5	1.503 (0.676–3.344)	0.317		
Hb				
≤ 148	Reference			
> 148	1.462 (0.839–2.546)	0.180		

*RS* Rad-score, *SII* systemic immune-inflammation, *NE* neutrophil, *Lym* lymphocytes, *Hb* hemoglobin, *EBV-DNA* Plasma Epstein–Barr virus DNA, *SUVmax* maximum standardized uptake.

### Performance and validation of the radiomics–clinical nomogram

**Figure 3:**
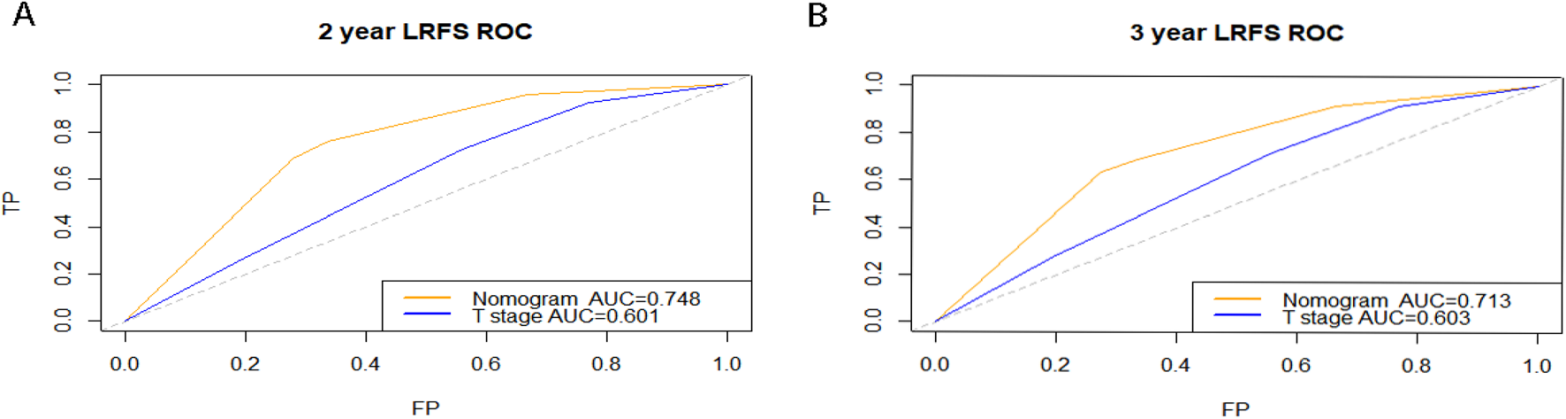
ROC curves for nomogram and T-stage for 2- and 3-year local failure-free survival in the training cohort.

**Figure 4:**
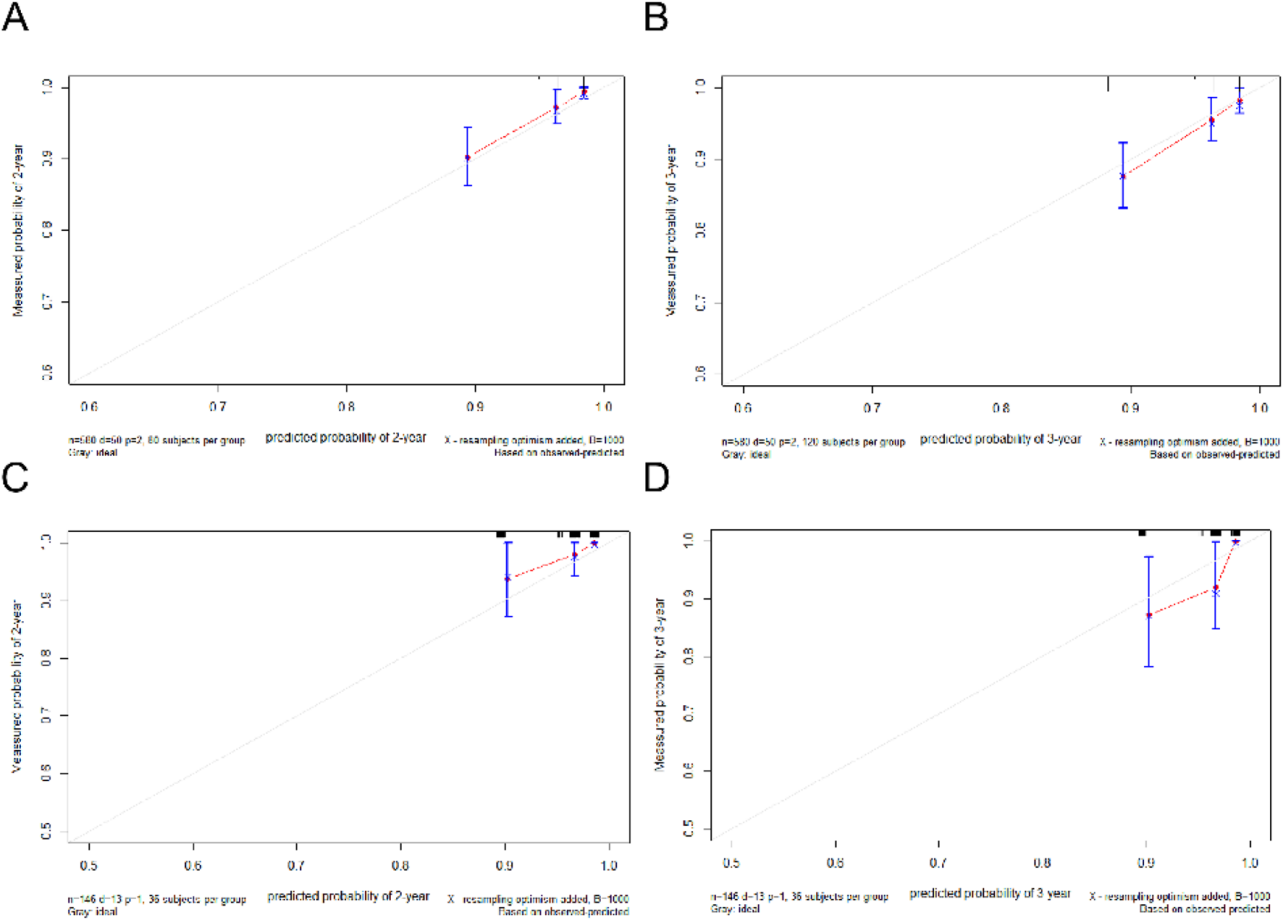
Calibration curves of the nomogram predicted and actually measured survival probabilities at 2 and 3 years of the training cohort (**A**,**B**) and validation cohort (**C**,**D**).

**Figure 5:**
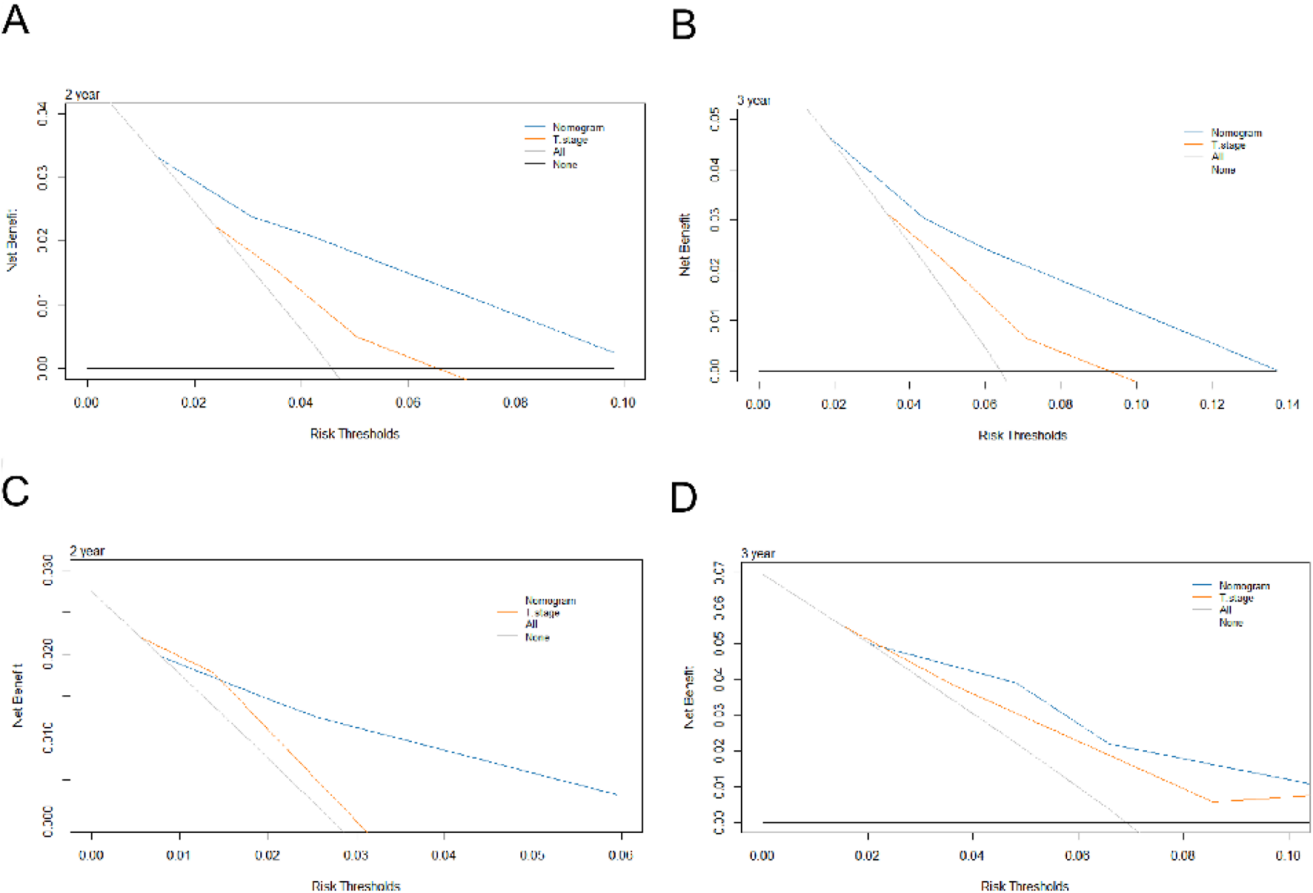
Decision curve analysis for 2- and 3-year survival predictions of the training cohort (**A**,**B**) and the validation cohort (**C**,**D**).

To identify the prediction performance of the nomogram, the C-index and AUCs in the training and validation cohorts were calculated. The C-index of the nomogram for LRFS was 0.71 (95% CI 0.67–0.74) in the training cohort and 0.70 (95% CI 0.64–0.76) in the validation cohort. The AUCs of the nomogram (0.75 for 2 years; 0.71 for 3 years) were found to outperform the 8th edition of the T-staging system (0.60 for 2 years; 0.60 for 3 years) in predicting 2- and 3-year LRFS (Fig. 3). The calibration curves for the probability of 2- or 3-year LRFS showed outstanding concordance between the estimation delivered by the radiomics–clinical nomogram and the actual observation in both training and validation cohorts (Fig. 4). Decision curve analysis (Fig. 5) revealed that the radiomics–clinical nomogram had significantly better net benefits than the 8th edition of the T-staging system across the range of reasonable threshold probabilities.

### Risk stratification

**Figure 6:**
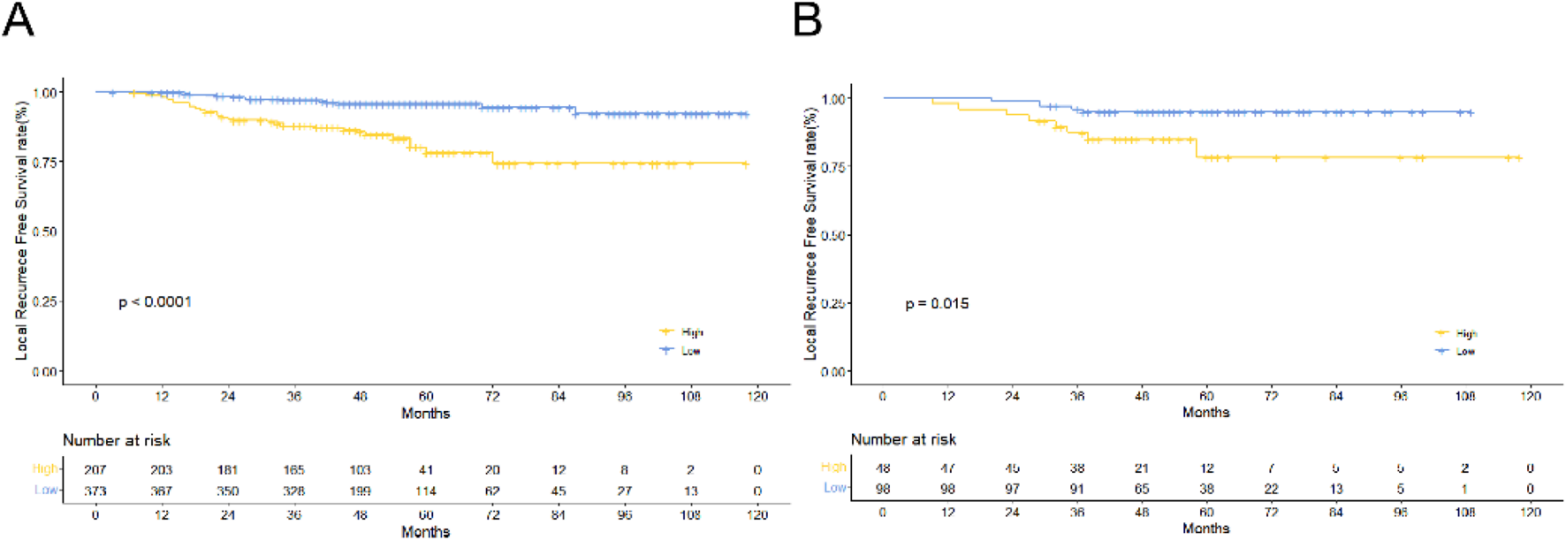
Kaplan–Meier survival curves for different risk groups of the training cohort (**A**) and the validation cohort (**B**).

To calculate the total nomogram score for each patient, the scores of all variables were added. All patients were further classified into high- and low-risk groups on the basis of the threshold (corresponding to a cut-off value of 72.9 points on the nomogram). The abovementioned cut-off value was identified using the ROC. The log-rank test and Kaplan–Meier curve revealed a significant difference between high- and low-risk patients’ local failure risk in the training cohort (*P* < 0.001), and a significant difference was also noted in the validation group (*P* = 0.015; Fig. 6). Consequently, these findings indicated that the radiomics–clinical nomogram could successfully identify patients at a higher or lower risk of local failure.

## Discussion

In this retrospective cohort study, we developed and validated a novel radiomics–clinical nomogram based on Rad-score and SUVmax to predict local recurrence in patients with NPC. Remarkably, our findings indicated that the nomogram could effectively categorize patients with significantly different probabilities of local failure into high- and low-risk groups and showed more robust predictive ability than the T-staging system.

Radiomics, a potential technique for obtaining crucial data on tumor heterogeneity, connects large-scale medical imaging information with clinical prognosis. Accordingly, a large amount of structural and functional information may be saved and could together contribute to predicting prognosis^12^. In addition, the detection of intratumor heterogeneity in particular patients can yield significant insights for therapeutic selection and drug development^13,14^. Many researches have revealed that radiomics features have excellent performance in predicting prognosis. Zhang et al. integrated a radiomics nomogram with the multi-parametric MRI-based radiomics features and the TNM staging system and reported superior prognostic accuracy in advanced NPC compared to the TNM staging system alone^15^.

Owing to the useful information it provides regarding tumor load and aggressiveness, 18F-FDG-PET/CT has been widely used to predict survival in patients with NPC. In a comparison of PET and CT radiomics for predicting local tumor control in HNSCC, Bogowicz et al. found PET to be more accurate than CT^16^. Therefore, greater focus should be placed on PET-based radiomics analysis for prognosis prediction. In the present study, we combined the advantages of radiomics and PET-CT to develop a prognostic model. Eight potential predictors from among 3375 radiomics features were extracted by LASSO regression and the Cox proportional hazards model, and Rad-score was built according to their coefficients. Rad-score showed a strong association with the risk of local failure and needs to be incorporated to construct a robust model. Moreover, some researches have also explored the local recurrence of NPC through radiomics analysis. Lv et al. found that combining PET and/or CT features with clinical parameters enhanced the predictive power compared to models with PET or CT radiomics features or clinical parameters alone, particularly in the local advanced subset^17^. Their model’s C-index was 0.69 (95% CI 0.59–0.77). This was consistent with our observations. The C-index in our study was 0.71 (95% CI 0.68–0.74); however, we had a considerably larger cohort than Lv et al.’s study (726 *vs*. 140) for the construction of predictive model.

As studies pursued the exploration of predictive factors of local recurrence, more and more potential factors were identified. According to Xiong et al.’s research, EBV-DNA was a promising predictor of survival in patients with locally advanced NPC^18^. Given the widespread use of 18F-FDG-PET/CT, SUVmax has often been acknowledged as a useful parameter for estimating the prognosis of patients with NPC^19,20^. We incorporated SUVmax, EBV-DNA, and other clinical variables in this study. SUVmax was the only significant clinical factor according to the findings of the multivariable Cox regression analysis. Several studies have examined the significance of SUVmax as a predictor of treatment response or outcome in patients with NPC^19,21,22^. In a study by Xie et al., the cut-off value for SUVmax of the primary tumor was observed to be 8.0, which is roughly consistent with our study (7.4), and both studies reported it to be correlated with local recurrence^19,23^. SUVmax is used to determine the degree of malignancy on the basis of the rate of fluorodeoxyglucose breakdown in tumors. A higher SUVmax indicates that the tumor is metabolically more active and is likely to show faster proliferation and have lower differentiation potential. Thus, SUVmax showed a significant correlation with LRFS in the present study.

Interestingly, the T-stage showed significance in univariate analysis but not in multivariate analysis. Although patients with T4 NPC may have a higher recurrence risk, the negative results of T-stage could be explained by collinearity in T-stage and SUVmax because both represented tumor burden. Lu et al. identified no correlation between T-stage and local recurrence^24^. Jiang et al. also reported that T-stage was not adequately effective in classifying patients into separate categories depending on the risk of local recurrence^25^. EBV-DNA was not enrolled into the nomogram owing to its lack of significance in univariate and multivariate analyses. We attribute this finding to the lack of stringent quality assurance measures implemented during clinical testing. Preiksaitis et al. showed that only 13/28 worldwide labs reported EBV-DNA levels within acceptable standards, demonstrating considerable interlaboratory variance. Variations in the perception of the detection threshold and the time of specimen collection after therapy restrict the use of plasma EBV-DNA detection in regular clinical applications^26^.

Numerous studies have focused on integrating multiple significant clinical factors into prognostic models. In the study of Lu, the clinical factors included age, sex, T-stage, and plasma EBV-DNA levels^24^. Zhang et al. developed a radiomics nomogram incorporating age, sex, N-stage, and Hb as clinical variables^27^. Zhao et al. also constructed a nomogram for OS that incorporated the variables of age, N-stage, sex, EBV-DNA, T-stage, NLR, LMR, LAR, and PNI^28^. In the present study, a radiomics–clinical nomogram was developed. This nomogram could effectively estimate the 2- and 3-year LRFS risk and was valuable for categorizing patients into high- and low-risk subgroups. Risk classification and identifying the high-risk individuals who are more likely to develop local recurrence could be of significant value for early clinical detection and timely intervention. Nomograms, which have been widely used to represent predictive models in cancer prognosis, are incredibly useful tools for clinical decision-making and are extremely valuable to both clinicians and patients. Nomograms can also facilitate personalized treatment based on the total score of significant variables. The allocation of proportions for different elements was determined based on the correlation coefficient in a rational manner. Furthermore, nomograms maximize the translation of complex regression equations into graphs, and the outcomes of predictive models are easier for patients to comprehend^28–30^.

Our research had some limitations. First, this investigation was conducted in an endemic region and a single hospital without external validation. Owing to the retrospective design of the study and a single data source, multicenter studies having larger cohorts with external validation are required to verify our results and improve the reliability of our radiomics study, thus enhancing the clinical application of prognostic signatures^31^. Second, our model still has scope for further improvement. The predictive model may perform better if the present model is integrated with genomics, thus expanding its knowledge so that it does not rely solely on spatial information. In addition, our algorithm may have scope for further enhancement by deep learning techniques. Finally, clinic personnel were relatively unfamiliar with the radiomics model and statistical analysis algorithms and found them to be quite complex. Practical tools, such as applications, should be developed in the future.

## Conclusion

In conclusion, we constructed a radiomics–clinical nomogram to predict local recurrence of NPC in patients by integrating radiomics features. Our model exhibited good prediction accuracy and independent discriminatory capacity in local recurrence-free survival. It may assist in personalized risk classification, facilitate individualized treatment strategies, and monitor clinical processes.

## Data Availability

Data are available upon reasonable request. The data sets generated during and analyzed during the current study are available from the corresponding author on reasonable request.
